# Medical and Psychological Risk Factors for Incident Hypertension in Type 1 Diabetic African-Americans

**DOI:** 10.4061/2011/856067

**Published:** 2011-08-23

**Authors:** Monique S. Roy, Malvin N. Janal, Alec Roy

**Affiliations:** ^1^Department of Ophthalmology, University of Medicine and Dentistry, New Jersey Medical School, Institute of Ophthalmology and Visual Science, 90 Bergen Street, Room 6164, Newark, NJ 07101-1709, USA; ^2^Department of Epidemiology and Health Promotion, College of Dentistry, New York University, 380 Second Avenue Suite 301, New York, NY 10010, USA; ^3^Psychiatry Service, New Jersey VA Health Care System, 385 Tremont Avenue, East Orange, NJ 07018, USA

## Abstract

*Objective*. To determine risk factors for the development of hypertension among African-Americans living with type 1 diabetes. *Methods*. African-Americans with type 1 diabetes (*n* = 483) participated in a 6-year followup. At both baseline and followup blood pressure was measured twice in both sitting and standing positions using a standard protocol. Patients had a structured clinical interview, ocular examination, retinal photographs, and blood and urine assays and completed the Hostility and Direction of Hostility Questionnaire (HDHQ) and the Beck Depression Inventory (BDI). *Results*. Of the 280 diabetic patients with no hypertension at baseline, 82 (29.3%) subsequently developed hypertension over the 6-year followup. Baseline older age, longer duration of diabetes, family history of hypertension, greater mean arterial blood pressure, overt proteinuria, increasing retinopathy severity, peripheral neuropathy, smoking, and higher hostility scores were significantly associated with the development of hypertension. Multivariate analyses showed that higher hostility scores and overt proteinuria were significantly and independently associated with the development of hypertension in this population. *Conclusions*. The development of hypertension in African-Americans living with type 1 diabetes appears to be multifactorial and includes both medical (overt proteinuria) as well as psychological (high hostility) risk factors.

## 1. Introduction

Persons living with diabetes have a prevalence of systemic hypertension twice as high as persons without diabetes [1–5]. Hypertension is an important problem in persons living with diabetes because it is strongly associated with the development of the major complications of diabetes—retinopathy, nephropathy, and cardiovascular disease [6–10]. As such, it is a major cause of morbidity and mortality among persons living with diabetes [11, 12]. Hypertension in persons living with diabetes appears to be more common in African-Americans than in whites [5]. For instance, data from the 1976–1980 National Health and Nutrition Examination Survey for mostly type 2 diabetic persons indicate that both systolic and diastolic blood pressures are higher in African Americans compared with whites at ages <54 years [5].

In persons living with diabetes, reducing morbidity and mortality from hypertension is predicated on identifying risk factors associated with the development of hypertension, so that treatment strategies may be implemented. In whites living with type 1 diabetes, medical risk factors reported in association with the development of hypertension include older age, longer duration of diabetes, male gender, smoking, poor glycemic control, and proteinuria [13]. Psychological factors have also been implicated in both the onset and progression of hypertension in the general population [14–17]. For example, in a meta-analysis of the relationship of personality to blood pressure, one of the strongest predictors of developing hypertension was found to be a high level of anger [15]. Furthermore, there is large literature suggesting that hostility and negative affective states, including depression, may be a risk factor for not only cardiovascular disease but also for hypertension [18–30]. One reason for the association of psychological factors with hypertension may be the exposure to chronic and environmental stressors, particularly for African Americans [17, 27, 31]. There is, however, no longitudinal study examining medical and psychological risk factors for the development of hypertension in a large group of African-Americans living with type 1 diabetes.

We had previously examined and subsequently carried out a 6-year followup of a large group of African-Americans living with type 1 diabetes, the *New Jersey 725* [8, 32, 33]. This 6-year followup provided an opportunity to fill the gap in knowledge about risk factors for the development of hypertension in the 280 African-American men and women with type 1 diabetes free of hypertension at baseline. The purpose of the present study was to examine putative medical and psychological risk factors in relation to the development of hypertension in our African-American diabetic cohort.

## 2. Methods

### 2.1. Study Population

Description of the original cohort has been previously reported [32]. Briefly, participants were identified from among 68,455 African-American patients discharged with a primary or secondary diagnosis of diabetes mellitus from 31 hospitals located in the seven counties lying within a 20-mile radius of the New Jersey Medical School, Newark. Approval by Institutional Review Boards at the University of Medicine and Dentistry of New Jersey, New Jersey Medical School, Newark, NJ, USA and at the various hospitals was first obtained. Subsequently, the medical record department at each hospital was given a list of potentially eligible patients and randomly retrieved patients' chart for review by the Principal Investigator (MSR), the study coordinator, and the two research assistants. From the review of 13,615 patient charts, 875 patients with an acute onset of diabetes before 30 years of age and on insulin therapy were found to be eligible. Patients of all ages were included in the study. Excluded were patients with type 2 diabetes, those diagnosed after age 30 whether on insulin or not, and patients with maturity-onset diabetes of youth [34, 35]. Of the 875 eligible patients, 725 (82.9%) were enrolled, 39 (4.4%) could not be traced, and 111 (12.7%) declined to participate [32].

Of the 725 patients, 508 (70.1%) participated in the 6-year follow-up examination, 44 (6.1%) could not be located, 34 (4.7%) refused examination, and 139 (19.2%) had died in the 6-year interval [8]. Excluding patients who had died since baseline, 508 of the 586 (87%) live patients had a 6-year follow-up. There was no contact with participants or data collection between the baseline and the 6-year examinations. At followup, 25 of the 508 (4.9%) participants were no longer receiving insulin and had not received a pancreas transplant. Since these 25 patients may not be truly insulin dependent, they have been excluded, leaving 483 (95.1%) of the 508 patients available for analysis. Comparison between patients on and off insulin at followup has been previously reported [8]. The mean (±SD) time of follow-up was 6.1 ± 0.5 years and median followup 6.0 years. Baseline characteristics of the 483 patients have been previously reported [8].

### 2.2. Procedures

Patients were examined in the Eye Clinic at University Hospital in Newark, NJ USA. On arrival, informed written consent was obtained. Similar procedures were obtained at both baseline and 6-year follow-up visits. Blood pressure was measured twice in both the sitting and standing positions using a random zero sphygmomanometer according to the Hypertension Detection and Follow-up Program protocol [36, 37]. The technician was trained according to a standardized protocol and was required to meet specified performance levels prior to certification [36]. Patients were sitting at rest for 10 minutes before any blood pressure measurement was made, and the two blood pressure measurements in either position were made at least 5 minutes apart. The averages of the two measurements in each position were used. Patients also underwent a complete eye examination and seven standard stereoscopic Diabetic Retinopathy Study retinal photographs [38]. Also obtained were height and weight. 

A structured clinical interview included detailed medical and ophthalmologic histories as well as sociodemographic factors and life-style variables (i.e., self-reported measures of smoking, alcohol consumption, and physical activity). Patients were asked whether their mother or father had hypertension for which they were treated. At baseline, patients ≥18 years of age completed the Beck Depression Inventory (BDI) [39]. The BDI is a 21-item self-report depression inventory which measures depressive symptoms [39]. For each item, there is a 1 to 4 score. The total score is obtained by adding up the scores on each of the 21 questions. 

At the second visit, patients ≥18 years of age completed the 51-item Hostility and Direction Hostility Questionnaire (HDHQ) [40]. Each item is answered as either “true” or “false”, and a scoring template is used to generate subscales scores which are summed to obtain a total hostility score. The HDHQ has been shown to have high validity and reliability [40]. 

Color fundus photographs obtained at both baseline and followup were graded for retinopathy severity in a masked fashion by the Wisconsin Fundus Photograph Reading Center in Madison, WI USA. The modified Early Treatment of Diabetic Retinopathy Study (ETDRS) Airlie House classification of retinopathy was used based on the severity level in the worse eye [41, 42]. Level 10 indicates no retinopathy, 20–35 minimal nonproliferative retinopathy, 43–53 moderate nonproliferative retinopathy and levels ≥61 severe proliferative retinopathy [42].

Venous blood was drawn for total glycosylated hemoglobin, using high-pressure liquid chromatography, and high- and low-density lipoprotein cholesterol (HDL-C and LDL-C) and total cholesterol using an enzymatic assay and separation spectrophotometry (Genzyme Diagnostics, Cambridge, MA USA). The normal range for total glycosylated hemoglobin is 4.2–7.0% and the intra-assay coefficient of variation 0.38–1.47%. A 4-hour timed urine collection was obtained for the measurement of albumin excretion rate (AER) and creatinuria using spectrophotometry (SmithKline Beecham Clinical Laboratory, Philadephia, PA USA). 

At both visits, patients' charts of previous hospital admissions and/or medical notes from private physicians were obtained and reviewed by the PI (MSR).

### 2.3. Definitions

Systemic hypertension was considered present if either the systolic pressure was ≥140 mm Hg and/or the diastolic ≥90 mm Hg, and/or the patient was taking antihypertensive medication [43]. When reviewing medical notes, patients' hypertension status and prescription of antihypertensive medications were confirmed. Antihypertensive medication prescribed by the patient's physician to only protect kidney function, as stated in the patients' medical chart, was not considered as antihypertensive medication. When the blood pressure measurements obtained in either the sitting or standing position differed in the characterization of the patient's hypertensive status as defined above, those measurements were discarded and the blood pressure measurements were repeated. The latter results were used to define the patient's hypertensive status. The 6-year incidence of hypertension was calculated from all patients (*n* = 280) who did not have hypertension at the baseline examination. Patient's age was the age at the time of baseline examination. Age at diagnosis was age at which diabetes was first recorded in the hospital record. Duration of diabetes was time between age at diagnosis and age at baseline. Microproteinuria was present if baseline AER was 20–200 *μ*g/min, and overt proteinuria if baseline AER was >200 *μ*g/min. A patient was considered depressed if at baseline the BDI score was ≥13. BDI cut-off scores of 12 to 14 have been found to have high predictive value as a screening instrument for depressive disorders in both the general population and in diabetic patients [44, 45]. 

Socioeconomic factors recorded included patient's level of education (for those ≥25 years of age), marital status, employment status, personal income (for those ≥18 years of age), and family income. Patient's socioeconomic status was classified from the Goldthorpe and Hope classification of occupations as middle-high (level 1–22) and lower (level 23–36) class using the occupation of the head of the household [46]. 

Alcohol abuse was considered present if the patient either currently reported drinking four or more alcohol drinks every day or had a past history of drinking four or more alcohol drinks a day every day for at least 1 year, as also documented from the review of the charts of all past hospital admissions. Smoking status was quantified as packyears calculated using the average number of cigarettes (or cigars) per day multiplied by the number of years the patient smoked until the baseline examination.

### 2.4. Statistical Tests

Data management and statistical analyses were performed using window-based statistical software (SPSS, Chicago, IL USA, version 17). Patients who did develop hypertension over the 6-year period (or who began using antihypertensive medication during that time, whether or not that treatment was effective), and those who did not were compared with regard to the various known and suspected risk factors. Mean levels of continuous variables were compared between groups with an independent samples *t*-test, and rates were compared with the *χ* ^2^ test. Odds ratios (ORs) and 95% confidence intervals (CIs) were also used to quantify the association between incident hypertension and relevant levels of each risk factor. The statistical significance of the associations was based on the Wald test. Rates are presented for each level of the risk factor, and the OR is computed with reference to the level thought to represent the lowest risk. Multivariate logistic regression analyses were used to identify independent risk factors for incident hypertension. Two risk factors were categorized for analysis because their distribution was skewed (smoking and BDI). Continuous values were used otherwise. 

Given the number of incident cases, we selected in the multivariate regression analysis five clinical variables (age, body mass index, smoking history, proteinuria, and family history of hypertension) on the basis of their importance in the literature in addition to the psychological variables (HDHQ and depression) [47]. The first rationale for including these predictors concerns the number of predictors that a regression can support [48]. Babyak suggests that regression modeling proceed with 10–15 incident cases per predictor. Limiting the number of predictors helps to avoid the influence of sampling variation that may be present, improving stability of the results and the likelihood that results will be replicable. The hostility variable was chosen because it represents a unique innovation of this study, and evaluating its independence from known risk factors (particularly depression) was a major goal of the multivariate analysis. The remaining variables were selected so as to maximize consistent reports in the literature; represent a range of causal mechanisms; modifiable over immutable risk factors; and proximal (with regard to hypertension) over more distal risks. All tests are 2-sided (where appropriate) and all use a 0.05 significance level.

## 3. Results

At baseline, the mean age of the 483 type 1 diabetic African-American patients (288 women and 195 men) was 27.5 ±  10.8 years and mean duration of diabetes 10.4 ± 8.6 years. At baseline, 203 (42.0%) of these patients already had hypertension and were excluded from further analysis. Over the 6-year followup, 82 of the 280 insulin-dependent patients [(29.3%) (95% CI, 24.0–34.9%)] without hypertension at baseline subsequently developed hypertension. At the 6-year followup, 60 patients were on one antihypertensive medication (26 patients on angiotensin-converting enzyme inhibitors (ACE), 6 on calcium channel blockers, 6 on beta-blockers, and 2 on diuretics), and 20 were on a combination of antihypertensive medications. Baseline characteristics of patients with and without hypertension at followup are presented in Table 1. 

### 3.1. Baseline Risk Factors for Hypertension

#### 3.1.1. Bivariate Analyses

The incidence of hypertension increased significantly with increasing age at baseline from 13.6% in those <10 years of age to 40.9% in those >30 years of age (test for trend, *P* < 0.01). The incidence of hypertension also increased with longer baseline duration of diabetes from 19.8% in patients with ≤5 years of diabetes to 40.0% in those with >20 years of diabetes duration (test for trend, *P* < 0.01). There was no significant association with gender.

The 6-year incidence of hypertension was significantly associated with baseline longer duration of diabetes (*P* = 0.02), more years of smoking (*P* = 0.008), a family history of hypertension (*P* = 0.04), higher mean arterial blood pressure (*P* = 0.001), overt proteinuria (*P* = 0.001), moderate to severe diabetic retinopathy (*P* < 0.001), peripheral neuropathy (*P* = 0.001), and a higher HDHQ score (*P* = 0.001) (Table 2). 

There was no significant association between developing hypertension and baseline body mass index (*P* = 0.46), insulin dose (*P* = 0.14), level of education (*P* = 0.30), socioeconomic status (*P* = 0.32), alcohol abuse (*P* = 0.09), blood total cholesterol levels (*P* = 0.23), total cholesterol/HDL-C (*P* = 0.16), glycosylated hemoglobin values (*P* = 0.11), or being depressed (*P* = 0.20).

#### 3.1.2. Multivariate Analyses

Multivariate logistic regression was used to evaluate the relative contribution of the baseline risk factors to the development of hypertension. Higher HDHQ scores and overt proteinuria were significantly and independently associated with the 6-year incidence of hypertension in this population (Table 3). This result was obtained whether or not depression was included in the model, suggesting that hostility makes a contribution to incident hypertension that is independent of depression (Table 4). The set of predictors produced a significant improvement in fit (*χ* ^2^  (14) = 33.0, *P* = 0.003) over an intercept-only model, and the Hosmer-Lemeshow statistic was consistent with good overall fit (*χ* ^2^ (8) = 9.7, *P* = 0.29). The model also showed good discrimination, as indexed by ROC analysis (AUC = 0.65, 95% CI = 0.56, 0.73); however, while there was excellent specificity (92.3%), sensitivity was low (36.7%). Interestingly, while age, family history of hypertension, and years of smoking showed significant bivariate associations with incident hypertension, these factors did not make an independent contribution. 

The analyses show a significant and independent association between baseline overt proteinuria and incident hypertension whether or not depression was included in the model (Tables 3 and 4). The association between proteinuria and hypertension suggests a causal pathway in which proteinuria precedes hypertension. To support this causal inference, we conducted a logistic regression analysis, otherwise parallel to that shown above, in which baseline hypertension was used as predictor of incident proteinuria. Results showed a statistically and clinically similar 6-year incidence of overt proteinuria in patients with (14.5%) or without (12.3%) hypertension at baseline, even after adjusting for family history of hypertension. Thus, while overt proteinuria is a risk factor for the 6-year incidence of hypertension, hypertension is not a risk factor for the 6-year incidence of overt proteinuria.

## 4. Discussion

In the present study, we found that a high percentage (29.3%) of 280 African-Americans with type 1 diabetes, who had no hypertension at baseline, subsequently developed hypertension over a 6-year follow-up period. Baseline psychological (high HDHQ score) and medical (overt proteinuria) risk factors were found to be independent predictors for the development of hypertension in this cohort.

In white persons with type 1 diabetes, the 10-year incidence of hypertension was also found to be a high (25.9%) by Klein et al., albeit a lower rate than the 6-year incidence of 29.3% in our type 1 diabetic African-Americans [13]. It has been similarly reported that African-Americans with type 2 diabetes have a higher risk for developing hypertension than their white type 2 counterparts [5]. The apparent higher risk of hypertension in African-Americans compared with whites, particularly in those with diabetes, is incompletely understood though it is noteworthy that the etiology of hypertension is generally thought to be multifactorial and to include medical, lifestyle, psychological, and genetic factors [13, 17, 31, 49].

To the best of our knowledge, risk factors associated with the development of hypertension have not been previously examined in a large cohort of African Americans living with type 1 diabetes. When we examined possible medical risk factors, we found that baseline overt proteinuria was the only independent medical risk factor associated with developing hypertension in our African-American patients. In type 1 diabetic whites baseline proteinuria was also reported to be an independent predictor for developing hypertension [7, 13, 49]. While the time relationship between hypertension and proteinuria is not always clear, our data indicate that in this cohort overt proteinuria precedes rather than follows the development of hypertension since patients with and those without hypertension at baseline have a similar risk of having overt proteinuria at the 6-year followup (see results) [50]. It is noteworthy that a high 37% of our African American patients with <5 years duration of diabetes at baseline had developed proteinuria at the 6-year follow-up [51]. Not only are African Americans living with type 1 diabetes at a particularly high risk for diabetic nephropathy, but they also develop this complication relatively early after diagnosis of diabetes [51]. Thus, evaluating kidney function early after diagnosis of diabetes, and regularly thereafter, is critically important in African Americans living with type 1 diabetes in order to reduce morbidity and mortality from the disease [9, 12, 51].

 Because African-Americans as a group are more likely to experience chronic sociocultural stressor, we were particularly interested in determining the respective role of psychological risk factors for hypertension [17, 27, 31, 52]. Our data show that a high hostility score on the HDHQ was significantly and independently associated with developing hypertension, even after adjusting for the traditional medical risk factors as well as for depression. While HDHQ and BDI measures showed significant levels of association (*r* = 0.37, df = 394, *P* < 0.001), that aspect of hostility which is related to incident hypertension does not appear to be related to depression, as depression was not associated with incident hypertension in this population (Table 3). Our data are in accord with a large literature suggesting that hostility may be a risk factor for cardiovascular disease and hypertension [14–31]. For example, Suarez and Williams showed that both men and women with high hostility scores on the Cooke Medley Hostility scale responded to a solvable anagram task—during which they were harassed—with greater diastolic blood pressure, greater forearm blood flow, and prolonged systolic blood pressure [23, 53]. They went on to demonstrate that high hostility individuals also had significantly enhanced and prolonged plasma norepinephrine and cortisol responses compared to low hostility subjects [54]. 

Also akin to the hostility findings of the present study is a 4-year follow-up study of 537 normotensive Finnish men [55]. In that study, each 1-point increase in anger-out on the Spielberger Anger Expression scale was associated with a 12% increase in the risk of developing hypertension [55]. Similarly, Bleil et al. found in 237 men with untreated hypertension that trait anger, anger temperament, and a propensity to express anger outwardly (anger-out) on the Spielberger scale were associated with heightened carotid atherosclerosis as measured by B-scan ultrasonography [56]. African-Americans may be more likely than whites to experience chronic sociocultural stressors and associated suppressed anger. For instance, in African-Americans living in high-stress areas in Detroit, suppressed anger was shown to be associated with elevated blood pressure [57].

In the present study, baseline years of smoking were significantly associated with the development of hypertension on bivariate analyses as previously reported in other studies [58, 59]. In a recent study, smoking in persons with mental illness was significantly associated with the development of hypertension [57]. Smoking is a known risk factor for cardiovascular disease, including coronary disease and stroke [60]. In this regard, it is noteworthy that smoking has been reported to increase arterial stiffness, promote atherosclerosis and inflammation, and lead to alteration in blood rheology [61]. However, smoking was no longer significant in the multivariate analyses because of its significant association with both the HDHQ score and proteinuria (data not shown). Similarly, age, family history of hypertension, and body mass index were not independent risk factors for incident hypertension because of their significant association with overt proteinuria (data not shown).

Strengths of the present study include that the sample is a large cohort of African-Americans living with type 1 diabetes; patients were followed for a mean of 6 years; both medical, lifestyle, and psychological risk factors were examined; a detailed medical history was obtained; standardized questionnaires were used to evaluate psychological factors; retinopathy severity was graded in a masked fashion on two separate occasions. 

Limitations of the present study include that we did not have African-Americans without diabetes serving as a control group. Furthermore, at the time of followup, 139 patients had died, and many patients included in the study were young. Therefore, the incidence of hypertension may be underestimated. Also blood pressure measurements were made on one single rather than on two separate visits, and there were no at-home or ambulatory measures of blood pressure [37]. Thus, the possibility of hypertension status misclassification exists. However, blood pressure was obtained by a certified technician using a standardized protocol and the random zero sphygmomanometer, a period of resting time was observed before and in between measurements, and measurements were repeated when there was a discrepancy in the results. Also, we used the cutoff score of ≥140 and/or ≥90 mm Hg for systolic and diastolic hypertension, respectively, which is higher than the target blood pressure of <130/80 mm Hg recommended for hypertensive patients with diabetes [37]. Due to time constraints, a battery of anger and anxiety measures was not administered. HDHQ scores measure trait rather than state hostility and thus are unlikely to be unduly impacted by diabetic complications. Finally, our patients were initially recruited from among patients admitted to hospital at some time during their life and may thus have more severe disease than persons living with type 1 diabetes but were never admitted to hospital. Thus, we cannot conclude that our findings can be generalized beyond the population that was studied. 

In summary, as far as we are aware, this is the first study reporting incidence rates of and risk factors for the development of hypertension in a large population of African-Americans living with type 1 diabetes. The results show that both medical risk factors—overt proteinuria—and psychological risk factors—high hostility score—may play a role. Thus, the results suggest the possibility of a multifactorial model for the development of hypertension in type 1 diabetic African-Americans. The potential impact of the findings in terms of clinical intervention might include the early identification of modifiable risk factors, which may be helpful in reducing the morbidity and mortality associated with diabetes.

## Figures and Tables

**Table 1:** Baseline characteristics of patients with and without hypertension* at the six-year followup.

	Hypertension*		
	Present	Absent	
	*N* ^†^ = 82	*N* ^†^ = 198	*P* ^‡^
	Mean ± SD		

Age (years)	27.0 ± 9.4	24.0 ± 10.0	0.02
Duration of diabetes (years)	10.3 ± 7.6	8.1 ± 7.9	0.03
Body mass index (kg/m^2^)	26.6 ± 8.3	25.0 ± 6.5	0.08
Glycosylated hemoglobin (%)	14.6 ± 4.6	13.6 ± 4.3	0.08
Total cholesterol (mg/dL)	213.4 ± 58.5	189.6 ± 40.9	<0.001
Systolic blood pressure (mm Hg)	116.5 ± 11.3	114.1 ± 10.6	0.09
Diastolic blood pressure (mm Hg)	78.1 ± 8.5	75.6 ± 8.6	0.04
Mean arterial blood pressure (mmHg)	86.5 ± 8.5	82.1 ± 9.2	<0.001
Beck Depression Inventory score	11.8 ± 9.0	9.5 ± 9.1	0.08
HDHQ score^§^	20.8 ± 9.1	17.1 ± 7.6	0.001

	*N * ^†^ (%)	*N * ^†^ (%)	*P* ^||^

Gender			0.89
male	31 (38.3)	74 (37.4)	
Proteinuria^¶^			0.001
none	48 (60.8)	152 (78.4)	
micro-	19 (24.1)	33 (17.0)	
overt	12 (15.2)	9 (4.6)	
Retinopathy severity^#^			<0.001
none	32 (39.5)	111 (56.1)	
minimal	25 (30.9)	74 (37.4)	
moderate	13 (16.0)	9 (4.5)	
severe	11 (13.6)	4 (2.0)	
Peripheral neuropathy			0.001
yes	45 (55.6)	67 (33.8)	
Stroke			0.77
yes	3 (3.7)	6 (3.0)	
Heart disease			0.34
yes	10 (12.3)	17 (8.6)	
Total Cholesterol/HDL-C** ≥4.5			0.15
yes	49 (73.1)	146 (81.6)	
Smoking			0.03
ever	43 (53.1)	77 (38.9)	
Family history of hypertension			0.05
yes	47 (59.5)	88 (46.6)	

*Systolic ≥140 and/or diastolic ≥90 mm Hg or use of antihypertensive medication; ^†^ *N *may vary due to missing data; ^‡^ *t*-test; ^ §^Hostility and Direction of Hostility Questionnaire [40]; ^||^chi-square test; ^¶^albumin excretion rate: none <20 *μ*g/min, micro- 20–200 *μ*g/min, overt >200 *μ*g/min;^**#**^Early Treatment of Diabetic Retinopathy Study severity level [42]; **high-density lipoprotein cholesterol.

**Table 2:** Six-year incidence of hypertension* by baseline characteristics: bivariate analysis.

Baseline characteristics	No. at risk^†^	Crude (%)	OR (95% CI)^‡^	*P* ^||^
Glycosylated hemoglobin (%)^¶^				0.11
<10.4	64	31.3	1.0	
10.41–13.45	74	23.0	0.66 (0.31–1.40)	
13.46–16.2	68	23.5	0.68 (0.31–1.46)	
>16.2	74	39.2	1.42 (0.70-2.87)	
Retinopathy severity				<0.001
none	143	22.4	1.0	
minimal	100	26.0	1.22 (0.67–2.21)	
moderate	22	59.1	5.01 (1.96–12.78)	
severe	15	73.3	9.54 (2.84–31.99)	
Proteinuria^#^				0.001
none	200	24.0	1.0	
micro-	52	36.5	1.82 (0.95–3.50)	
overt	22	59.1	4.57 (1.84–11.36)	
Peripheral neuropathy				0.001
no	167	21.6	1.0	
yes	113	40.7	2.50 (1.48–4.23)	
Total cholesterol/HDL-C**				0.16
<4.5	196	25.5	1.0	
≥4.5	51	35.3	1.59 (0.83–3.08)	
HDHQ score^¶††^				0.001
0–12	71	18.3	1.0	
13–16	51	27.5	1.69 (0.71–3.99)	
17–23	62	30.6	1.97 (0.88–4.42)	
≥24	63	44.4	3.57 (1.64–7.79)	
BDI score ≥13**^‡‡^**				0.20
no	159	27.7	1.0	
yes	63	36.5	1.50 (0.81–2.79)	
Smoking (packyears)^¶^				0.008
0	160	24.4	1.0	
<5	44	29.5	1.34 (0.64–2.81)	
5–14	40	32.5	1.53 (0.72–3.26)	
≥15	36	47.2	2.85 (1.35–6.03)	
Body mass index (Kg/m^2^)				.46
<25	153	27.5	1	
>25	127	31.5	1.22 (0.73–2.04)	
Duration of diabetes (years)^¶^				.02
<5	111	20.7	1	
5–9	69	36.2	2.17 (1.10–4.26)	
10–17	57	28.1	1.49 (0.71–3.12)	
≥18	43	41.9	2.76 (1.29–5.89)	
Family history of hypertension				.04
no	133	24.1	1	
yes	136	35.3	1.72 (1.01–2.93)	
Mean arterial blood pressure (mmHg)^ ||||^				.001
<82.2	113	23.9	1	
82.3–90.5	105	22.9	0.94 (0.50–1.77)	
>90.5	62	50.0	3.19 (1.65–6.16)	
Education				0.32
≤high school	75	37.3	1	
≥college	71	29.6	0.71 (0.35–1.41)	
Socioeconomic status				0.30
middle-high	133	26.3	1	
lower	147	32.0	1.32 (0.78–2.21)	
Alcohol abuse				0.09
no	240	24.6	1	
yes	19	42.1	2.23 (0.86–5.81)	

*Systolic ≥140 or diastolic ≥90 mm Hg or antihypertensive medication; ^†^number at risk may vary due to missing data; ^‡^odds ratio (95% confidence interval); ^||^test for trend, using Cochran Armitage trend test; ^¶^quartiles; ^#^albumin excretion rate: none <20 *μ*g/min; micro- 20–200 *μ*g/min; overt >200 *μ*g/min; **high-density lipoprotein cholesterol; ^††^HDHQ: Hostility and Direction of Hostility Questionnaire score [40]; **^‡‡^**Beck Depression Inventory score ≥13 at visit 1 [39]; ^||^tertiles.

**Table 3:** Six-year incidence of hypertension* by baseline characteristics: multivariate analysis.

Characteristic	OR (95% CI)^ †^	*P* ^‡^
Age (per year)	0.99 (0.95–1.04)	0.88
Body mass index (per Kg/m^2^)	1.02 (0.98–1.07)	0.37
Smoking (packyears)^ §^		0.67
0	1.0	
<5	0.98 (0.43–2.24)	
5–14	1.20 (0.50–2.89)	
≥15	2.04 (0.74–5.62)	
Family history of hypertension^||^	1.63 (0.87–3.05)	0.13
Proteinuria^¶^		0.16
none	1.0	
micro-	1.39 (0.63–3.09)	
overt	3.00 (1.03–8.73)	
HDHQ score	1.06 (1.02–1.10)	0.001

*Systolic ≥140 or diastolic ≥90 mm Hg or antihypertensive medication; **^†^**odds ratio (95% confidence interval); ^‡^ *P* values; ^§^quartiles; ^||^present versus absent; ^¶^microalbuminuria versus none and overt proteinuria versus none.

**Table 4:** Six-year incidence of hypertension* by baseline characteristics: multivariate analysis.

Characteristic	OR (95% CI)^ †^	*P* ^‡^
Age (per year)	1.00 (0.95–1.05)	0.99
Body mass index (per Kg/m^2^)	1.04 (0.99–1.09)	0.09
Smoking (packyears)^ §^		0.51
0	1.0	
<5	0.71 (0.27–1.89)	
5–14	0.89 (0.34–3.32)	
≥15	1.93 (0.66–5.64)	
Family history of hypertension^||^	1.54 (0.78–3.04)	0.21
Proteinuria^¶^		0.05
none	1.0	
micro-	1.74 (0.73–4.19)	
overt	3.75 (1.26–11.16)	
HDHQ score	1.08 (1.03–1.13)	0.002
BDI score ≥13**	0.64 (0.29–1.43)	0.28

*Systolic ≥140 or diastolic ≥90 mm Hg or antihypertensive medication; **^†^**odds ratio (95% confidence interval); ^‡^ *P * values; ^§^quartiles; ^||^present versus absent; ^¶^microalbuminuria versus none and overt proteinuria versus none; ^ #^Hostility and Direction of Hostility Questionnaire score [40]; **Beck Depression Inventory score ≥13 at visit 1: depressed versus not depressed [39].

## Abbreviations

AER: Albumin excretion rate
BDI: Beck Depression Inventory
CI: Confidence interval
HDHQ: Hostility and Direction of Hostility Questionnaire
OR: Odds ratio
SD: Standard deviation.
